# Transforming fire governance in British Columbia, Canada: an emerging vision for coexisting with fire

**DOI:** 10.1007/s10113-022-01895-2

**Published:** 2022-03-22

**Authors:** Kelsey Copes-Gerbitz, Shannon M. Hagerman, Lori D. Daniels

**Affiliations:** Faculty of Forestry, University of British Columbia, 3041-2424 Main Mall, Vancouver, BC V6T 1Z4 Canada

**Keywords:** Transformation, Governance, Wildfire, Indigenous fire, Command and control

## Abstract

The dominant command and control fire governance paradigm is proven ineffective at coping with modern wildfire challenges. In response, jurisdictions globally are calling for transformative change that will facilitate coexisting with future fires. Enacting transformative change requires attention to historical governance attributes that may enable or constrain transformation, including diverse actors, objectives, worldviews of fire, decision-making processes and power, legislation, and drivers of change. To identify potential pathways for transformative change, we systematically examined the history of fire governance attributes in British Columbia (BC), Canada (until 2020), a region that has experienced seven catastrophic fire seasons in the twenty-first century. By reviewing 157 provincial historical documents and interviewing 19 fire experts, we delineated five distinct governance eras that demonstrated the central role of government actors with decision-making power shaping fire governance through time, superseding First Nations fire governance starting in the 1870s. The emerging vision for transformation proposed by interviewees focuses on the need for increased decision-making power for community actors, yet legacies of entrenched government power and organizational silos between fire and forestry continue to constrain transformation. Although progress to overcome constraints has been made, we argue that enabling transformative change in fire governance in BC will require intervention by the provincial government to leverage modern drivers of change, including recent catastrophic fire seasons and reconciliation with First Nations.

## Introduction

Globally, twenty-first century fire seasons are revealing the inadequacy of conventional “command and control” (Holling and Meffe 1996) fire governance models that rely on reactive fire management (Smith et al. 2016; Steelman 2016; Nowell and Steelman 2019; Tedim et al. 2019). Many now argue that transformative change is needed to develop new models that facilitate proactively coexisting with fire (Moritz et al. 2014; Higuera et al. 2019; McWethy et al. 2019; Tedim et al. 2019). These calls for transformation are catalyzed by fires that increasingly threaten human lives and livelihoods (Bowman et al. 2013), which are exacerbated by fire exclusion policies (Bowman et al. 2011) and climate change (Jolly et al. 2015). While new fire governance models have been proposed (Steelman 2016; Tedim et al. 2019), existing research has yet to adequately consider how potential transformative changes may be enabled or constrained by legacies of historical fire governance.

Understanding transformation in fire governance requires attention to history because modern environmental challenges are inevitably rooted in the processes and outcomes of past decision-making (Offen 2004; Mathevet et al. 2015). In western North America, for example, historical policies of fire suppression and exclusion imposed by colonial governments (and contested by Indigenous peoples) have today resulted in a build-up of hazardous fuels, an increased fire risk, and decreased forest resilience (Stephens et al. 2013; Hessburg et al. 2019). Furthermore, these historical policies interrupted Indigenous fire stewardship (Eriksen and Hankins 2014; Lake and Christianson 2019) that historically maintained biodiversity (especially of woody and non-woody plants) and habitat heterogeneity (Hoffman et al. 2021). Tracking how governance has, and has not, changed through time can therefore help identify opportunities to ensure that transformation is both equitable and ecologically meaningful (Offen 2004; Davis 2009).

Governance, as it is used here, is defined as attributes that influence environmental outcomes, including organizations or individual actors, objectives, legislation, decision-making processes and power, and worldviews (Lemos and Agrawal 2006; Bennett and Satterfield 2018). Governance both shapes and arises from environmental outcomes as these attributes interact through time (Mathevet et al. 2015). For example, centralized government (often colonial) actors wielding more power tend to dictate objectives and legislation, often resulting in landscapes that no longer support community objectives (Lemos and Agrawal 2006; Armitage et al. 2012; Cockerill and Hagerman 2020). In this case, communities are both excluded from governance yet are affected by and respond to the environmental outcomes, often demanding more decision-making power as a result. A governance transformation could thus occur when undesirable environmental outcomes (such as catastrophic impacts from wildfire) prompt a revisioning and intentional shift in key governance attributes (Westley et al. 2013; Chaffin et al. 2016), but only if constraints are recognized and addressed.

A primary constraint on governance transformations is institutional or organizational rigidity (Farrelly and Brown 2011; Chaffin et al. 2016). Organizational rigidity can be understood as a manifestation of the path-dependency of entrenched power (Offen 2004; Westley et al. 2011; Chaffin et al. 2016). In the case of the United States Forest Service (a primary decision-maker on federal land), for example, organizational rigidity caused by broad social-political dynamics and decision-making by individual actors perpetuated the status quo, despite changes to formal and informal policies (Moseley and Charnley 2014; Schultz et al. 2019). This rigidity was especially problematic because it was entrenched within two siloed sub-organizations (fire and forestry) with different expertise and objectives and led to different visions for the future of fire (Schultz et al. 2019).

One common proposal for overcoming organizational rigidity is the redistribution of decision-making power to community-based actors (Jasanoff and Martello 2004; Offen 2004; Davis 2009; Armitage et al. 2012). This redistribution is also a primary attribute of proposed models of governance for coexisting with fire that recognize the current command and control model is often inequitable (Steelman 2016; Kelly et al. 2019; Tedim et al. 2019). However promising, transformative change to achieve these models is unlikely to be successful in practice without an historically informed understanding of current governance attributes. In particular, examining the distribution of power is imperative (Schultz et al. 2019; Tedim et al. 2019), as failing to do so risks perpetuating (rather than redressing) historical and ongoing environmental injustices (Agrawal et al. 2008), such as those experienced by Indigenous peoples who are often marginalized from decision-making but bear an outsized burden of impacts from catastrophic fire events (Eriksen and Hankins 2014; Lake and Christianson 2019; Erni et al 2021).

### Study context

Fire governance is understudied in Canada, where command and control governance relies primarily on reactive fire suppression objectives managed by provincial governments (McGee et al. 2015; Tymstra et al. 2020). Catastrophic fire seasons are increasingly exceeding fire suppression capacities, especially under climate change (Stocks and Martell 2016; Wotton et al. 2017; Tymstra et al. 2020). This fire governance model results in thousands of evacuations annually (Beverly and Bothwell 2011), many of which disproportionately affect Indigenous communities (Christianson 2015; McGee et al. 2019; Zahara 2020; Erni et al. 2021). In one case, catastrophic fire seasons in 2017 and 2018 in the western province of British Columbia (BC) prompted calls for new fire governance that prioritizes proactive fire objectives and redistributes decision-making power to Indigenous and local communities (Abbott and Chapman 2018; Sankey 2018).

**Fig. 1:**
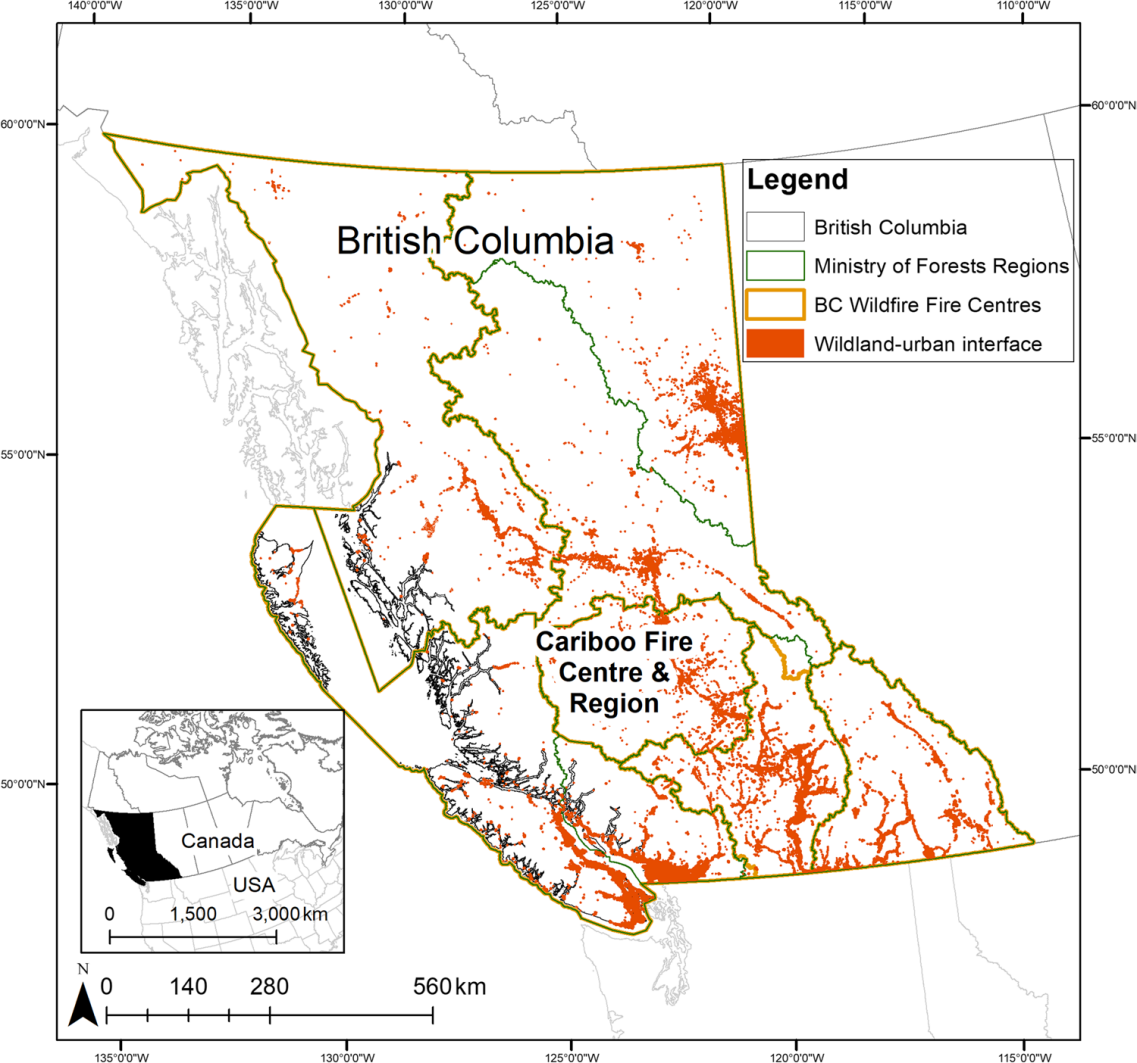
Fire governance in British Columbia, Canada, including Fire Centers (jurisdiction of the BC Wildfire Service), Forest Regions (jurisdiction of the Ministry of Forests), and the wildland-urban interface (2.75 km buffer around an area with ≥ 25 structures per hectare) where community values are most at risk from fire. Inset map is the location of British Columbia

Current governance authority over BC’s 95.2 million hectares — all of which is adapted to periodic fire (Wong et al. 2003; Hoffman et al. 2017) — is contested between First Nations^1^ and the government (Caverley et al. 2020). Across ~ 95% of BC, treaties with First Nations were never negotiated and today they still declare sovereignty over their unceded lands (Borrows 2017; Wilson 2019). Since time immemorial, First Nations have stewarded the lands using fire under their own governance models (Gottesfeld 1994; Lewis et al. 2018; Lake and Christianson 2019; Verhaeghe et al. 2019). The colonial land governance system, imposed when BC became a province in 1871, now classifies ~ 94% of land as provincial “Crown land,” of which about two-thirds is forested. The remainder is private (4.9%), federal Crown (1%), and First Nations Treaty and Title (0.2%) lands (MFLNRO 2011). Despite contested land sovereignty, the provincial government holds decision-making authority. As part of the provincial government, the BC Wildfire Service^2^ is mandated to oversee wildfire management^3^ on provincial and federal Crown land, local government land on request of the local government, Treaty Settlement lands, and private lands. The BC Wildfire Service is divided into six Fire Centers (Fig. 1), which are roughly contiguous with the Ministry of Forests, Lands, Natural Resource Operations, and Rural Development (hereafter, Ministry of Forests^4^) Regions.

**Fig. 2:**
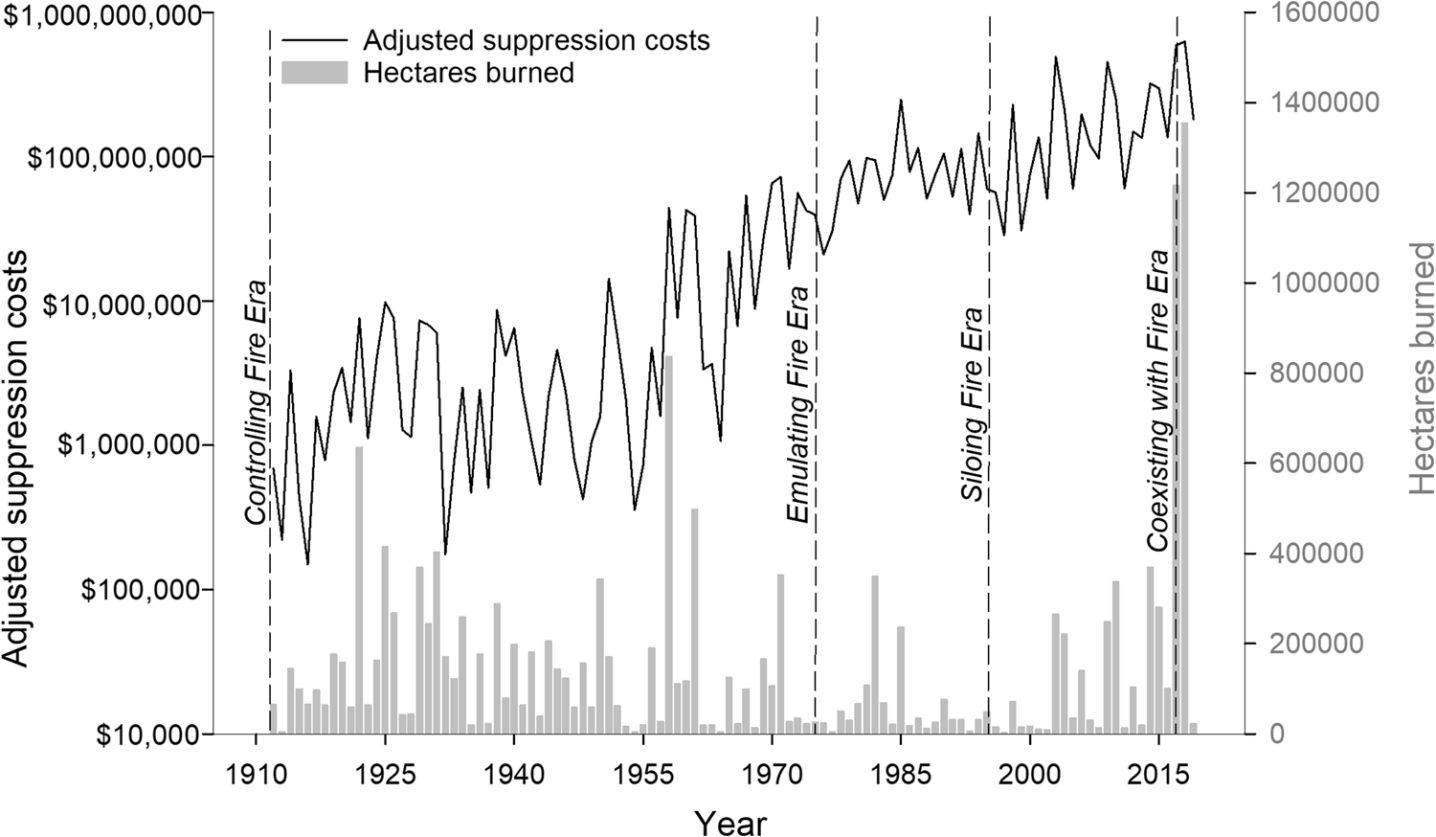
Adjusted (for inflation to 2020) fire suppression costs in Canadian dollars (black line) and hectares burned (grey bars) in British Columbia from 1912–2019. Data from BC Wildfire Service Annual Reports were compiled and provided by John Parminter and Arial Eatherton. Note logarithmic scale on left *y*-axis

Between 1998 and 2021, ~ 5.7 million ha (~ 14 million acres) burned in BC, including seven significant wildland-urban interface (WUI) fires that forced evacuation of ~ 125,000 people (Public Safety Canada 2021). Total suppression costs during these six fire seasons were over $2.6 billion CAD (adjusted for inflation to 2020 dollars; Fig. 2), accounting for over one-third of suppression costs since records began in 1912 (BC Wildfire Service 2021). Total indirect costs to livelihoods are much higher (Sankey 2018; Johnston et al. 2020). The 2017 fires burned a record area of 1.2 million ha (surpassed in 2018 when 1.35 million ha burned), of which ~ 73% was in the Cariboo Fire Centre (hereafter, the Cariboo). Until COVID-19, the 2017 fire season prompted the longest provincial state of emergency lasting 70 days, during which 65,000 people were evacuated, including 26 First Nations communities (Abbott and Chapman 2018).

The aims of this study are to track historical fire governance attributes in BC, identify key factors driving change, and understand how historical governance attributes may enable or constrain transformative change. The governance attributes of interest include actors, decision-making power, objectives, strategies to achieve objectives, legislation, and worldviews of fire. While the primary scale of interest is the province of BC and the Cariboo, we consider how governance attributes and drivers of change interact across broader (Canadian) and finer (local) scales through time.

## Methods

We used a case study approach (Creswell 2013) to address three specific questions: (1) How has fire governance in BC changed through time? (2) What drivers have shaped fire governance through time? (3) What do fire experts envision for the future of fire governance in BC and to what extent do current governance attributes support this vision? Our analyses draw on two sources of data: (1) historical documents and (2) semi-structured interviews.

We analyzed all documents written by provincial or federal authorities with stated objectives on fire in BC from 1874 to 2020 (*n* = 157 documents; 13,339 pages total). These included provincial forestry reviews (*n* = 7); annual reports (*n* = 110) and strategy documents (*n* = 5) of the BC Wildfire Service; research reports of the provincial forestry research program (*n* = 36); provincial fire season reviews (*n* = 2); federal forestry or fire strategy documents (*n* = 4); and provincial fire legislation (*n* = 4). We recorded the stated purpose and source of each document, acknowledging that all documents are social products with inherent biases (Coffey 2013), and that our sample did not include non-government perspectives (e.g., First Nations, local communities, forest industry) (Bowen 2009; Coffey 2013).

Complementing the document analysis, we conducted 19 semi-structured interviews (Holstein and Gubrium 1995; Schensul et al. 1999) with fire experts between 2019 and 2020. Interviews with fire experts in BC explored historical attributes and visions for the future of fire governance in BC. Respondents included fire or forestry practitioners within provincial government organizations (the Ministry of Forests or BC Wildfire Service) or in roles directly related to fire outside government (e.g., consultants or other non-governmental organizations, hereafter, NGOs). Interviewees were selected through a combined stratified purposeful and snowball sampling approach (Palinkas et al. 2015) until saturation of key themes was met (Small 2009). Interviewees included 11 provincial experts, of which five represented NGOs. Eight Cariboo regional experts were also interviewed to capture experiences during the 2017 fire season, including interviewees affiliated with the BC Wildfire Service (four), Ministry of Forests (three), and an NGO (one). Interviews were audio-recorded with permission and transcribed for a total of 328 pages. To preserve confidentiality where requested, interviewees are referred to by number (e.g., Expert #1); otherwise, interviewees are referred to by their affiliation and a number (e.g., BCWS #2).

Systematic and iterative coding of historical documents and interviews was undertaken in NVivo software (12.6.0 2020) using a combined deductive and inductive approach (Coffey 2013). Deductive coding tracked the general suite and relative importance of the governance attributes of interest through time. The inductive coding conducted simultaneously identified emergent themes, such as the governance connection between fire and forests and different scales and drivers of change. Finally, we delineated five fire governance eras based on periods of stability and key moments of change defined by broad scale shifts in the suite of governance attributes.

## Fire eras in British Columbia

**Fig. 3:**
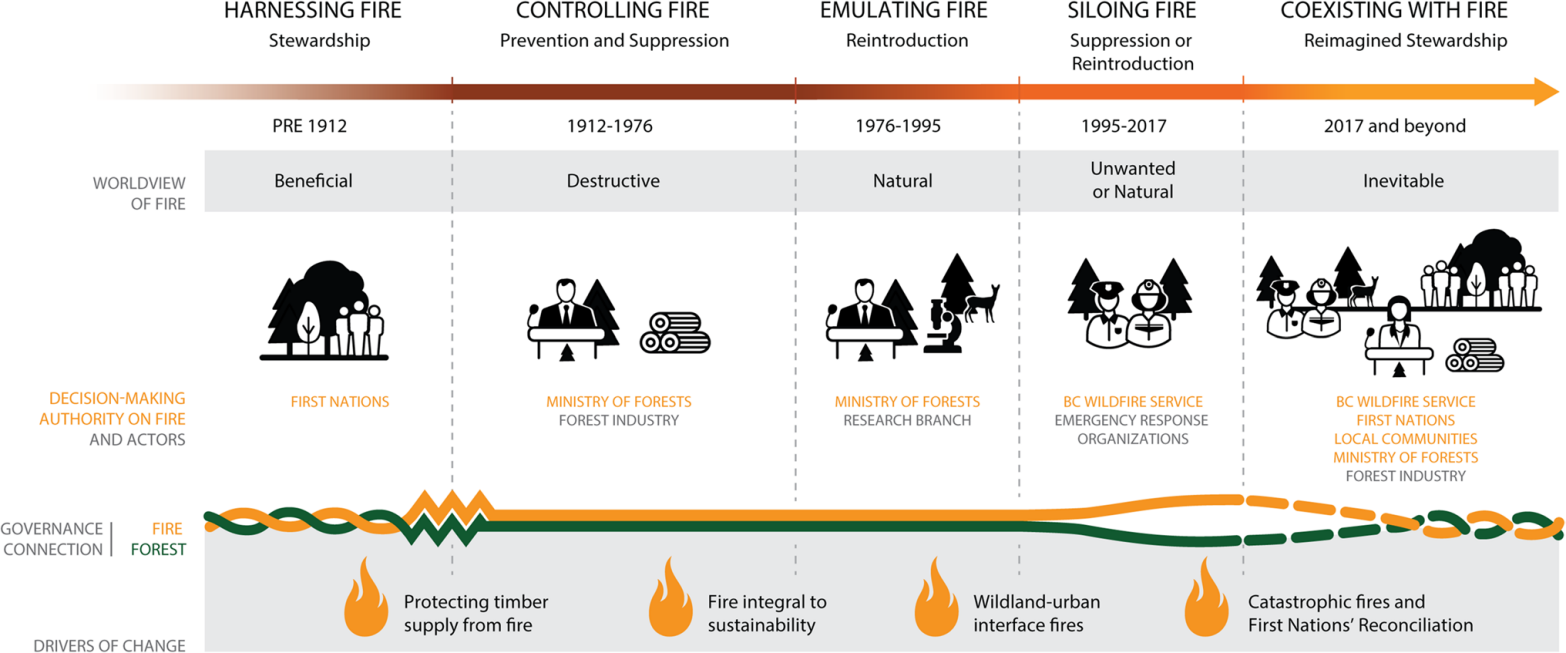
Fire governance eras in British Columbia which were distinguished based on worldview of fire, actors with decision-making authority on fire, governance connections between fire and forestry, and key drivers of change between eras. Note the First Nations’ decision-making authority and intertwined governance connection required for coexisting with fire in the *Coexisting with Fire* era

Since 1912, the BC provincial government has been the primary decision-making authority that implements objectives, strategies, and legislation around fire. Embedded in these governance attributes is the worldview of fire as destructive to timber, in contrast to the worldview of fire as beneficially held by First Nations practicing fire stewardship for millennia prior (Lake and Christianson 2019; Hoffman et al. 2022). In response to the 2017 and 2018 fire seasons, experts and recent documents consistently described an emerging vision for coexisting with fire that would require transformative change to incorporate the worldviews and objectives of diverse actors. However, our results show that rigidity and siloed expertise in the provincial fire and forestry organizations are key constraints on transformative change. We present our analysis in five distinct governance eras characterized by different actors with decision-making authority, worldviews and objectives of fire, and governance connection between fire and forests (Fig. 3).

### Harnessing fire (pre-1912)

Prior to the formalization of colonial authority over fire in the 1912 BC Forest Act, First Nations actors harnessed fire under their own systems of governance for a variety of cultural, spiritual, and ecological objectives. These systems of governance reflected a worldview of fire as beneficial for biodiversity and cultural continuity (Turner et al. 2000; Huffman 2013; Lewis et al. 2018; Lake and Christianson 2019; Hoffman et al. 2021), although experiences with fire varied and burning practices were not universal (Lake 2013). Referring to practices prior to 1912, the Ministry of Forests noted that First Nations “*burned the forest every year to ‘light the salmon up the Fraser River*,’ *as well as to improve hunting*” (Province of BC 1914, pg. 82). Early settlers similarly held the worldview that “*fire was more beneficial than otherwise*” (Whitford and Craig 1918, pg. 126) but used it for different objectives than First Nations (Hoffman et al. 2022). Settlers perceived it as a “*natural accompaniment to the routine of progress and development*” (Fulton 1910, pg. 59) and intentionally set fires for land clearing and agriculture.

In contrast to early settlers, federal and provincial government actors sought progress by denying the benefits of First Nations’ fire stewardship in colonial documents. The colonial BC government began to institutionalize their worldview of fire as destructive to timber by passing the Bush Fire Act in 1874, which focused on fire prevention objectives through strategies such as financial penalties for setting fire and prohibiting burning except by permit (Parminter 1981). Although the Bush Fire Act did not apply to the entire province until 1887, it marked the transition towards a colonial system of fire governance that superseded First Nations fire governance in BC, mirroring colonial suppression of Indigenous fire across North America (Murphy et al. 2007; Lake and Christianson 2019; Nikolakis and Roberts 2020). This transition was reinforced by the establishment of Indian Reserves and Residential Schools by the federal Indian Act, pre-emption policies restricting First Nations’ access to land, and the smallpox epidemic that killed up to two-thirds of First Nations peoples in BC (Caverley et al. 2020).

As timber emerged as “*the most valuable asset in the hands of the Government*” (Fulton 1910, pg. 67), the colonial government objective of fire control became “*the supreme need of [BC’s] forests*” (Fulton 1910, pg. 60). At the end of the *Harnessing Fire* era, the Bush Fire Act “*lacked an adequate organization for enforcement*” and the fire wardens who held decision-making authority for fire control were considered “*ineffective and subject to…ridicule*” because of their limited access and numbers (Whitford and Craig 1918, pg. 126). Recognizing this inefficiency, and driven by colonial worldviews of fire, the Royal Commission of Inquiry on Timber and Forestry in 1910 advocated for a single fire objective, stronger legislation, and a new organization to enforce it:

*we have in mind the active* ***prevention**** of fire by the systematic work of a well-knit [government] organization…That the timber, upon which our whole future as a lumber-producing country depends, should be left…under the imminent menace of fire…is so absurd…that regulation is imperative (Fulton pg. 65, emphasis in situ).*

Despite First Nations harnessing the benefits of fire for millennia, colonial actors, worldviews, and legislation introduced in 1912 forcibly limited First Nations fire stewardship into the twentieth and twenty-first centuries (Lake and Christianson 2019).

### Controlling fire (1912–1975)

The *Controlling Fire* era cemented colonial fire governance through the creation of the Ministry of Forests by the Forest Act in 1912. The Forest Act enshrined the worldview of fire as a “*common enemy*” of timber industry actors who were the “*commercial backbone*” of BC (Province of BC 1914, pg. 57). Because of the “*perceived – real or not – timber shortage*” (NGO #14), fire control became the central objective for ensuring continued timber supply (and protection of new communities) through the “***prevention*** *of forest fires [and] the* ***fighting*** *of those that have been allowed to spread*” (Fulton 1910, pg. 59, emphasis in situ). Three interrelated objectives — fire control through prevention and suppression — dominated this era and reflect the command and control fire governance model that prevailed across colonial North America (Smith et al. 2016; Minor and Boyce 2018; Nowell and Steelman 2019).

To achieve fire control objectives, the Ministry of Forests required new legislation and involved new actors: “*to be effective, a forest protection service must be supported by [1] comprehensive legal authority [and 2] close co-operation of all the allied interests*” (Whitford and Craig 1918, pg. 129). The 1912 Forest Act legally mandated the Ministry of Forests, who oversaw the BC Wildfire Service, to control fire on provincial Crown land (Parminter 1981). The Forest Act also gave the Ministry of Forests authority to enforce fire prevention strategies such as financial penalties and jail for people who “*wilfully set fire*” without a burn permit or refused to provide firefighting assistance (Province of BC 1914, pg. 90). These strategies disproportionately affected First Nations, who attempted to continue their fire stewardship practices but were forced to uphold colonial worldviews and objectives (Christianson et al. 2013; Eriksen and Hankins 2014). For industry actors, the Forest Act mandated broadcast burning in the Vancouver Forest District, a universal fire prevention strategy used to minimize the “*slash evil*” left after logging (Province of BC 1913, pg. 13). By the end of this era, however, the use of this prevention strategy waned in the Vancouver District until the Report of the Commissioner (Sloan 1945) recommended that it no longer be compulsory related to industry concerns over cost and liability.

To ensure “*co-operation of all the allied interests*,” (Whitford and Craig 1918, pg. 129), the Ministry of Forests relied on “propaganda” to raise awareness for fire prevention objectives: “*The number of fires started by [the public] is still far too high, but…we may confidently assume that our propaganda is bearing fruit*” (Province of BC 1920, pg. 23). This propaganda was developed to remind people of their “*obligation to assist the Government in preventing fires*” (Province of BC 1914, pg. 90) and evolved from fliers and radio advertisements to signage on highways throughout the era. Combined, these strategies further engrained the colonial worldview of fire as destructive to the broader public.

The BC Wildfire Service was the actor primarily responsible for the fire suppression objective once ignitions occurred. They desired to be as follows:

“*an organization so equipped and manned that every fire is spotted immediately [when] it starts and is extinguished… Every holocaustic conflagration is, in its incipiency, small enough to be crushed beneath a man's heel*” (Sloan 1945, pg. 131).

To successfully “crush” every fire, two key strategies were used: a fire suppression force and equipment (Parminter 1981), colloquially (and paternalistically) referred to as “*the boys and the toys*” (NGO #11; BCWS #13). The “boys,” or fire suppression personnel of the BC Wildfire Service, were embedded in the Ministry of Forests at the sub-regional (district) level. The “toys” were developed in collaboration with research-based actors such as the Canadian Forest Service, and advances in this era included the province-wide weather network that supported the Canadian Forest Fire Danger Rating System (Stocks et al. 1989; Coogan et al. 2020) and the aerial attack fleet that was implemented after proving its utility during World War II. Although the Ministry of Forests continued to promote the worldview of fire as destructive throughout this era, by the early 1970s, an alternative worldview of fire as natural began to emerge.

### Emulating fire (1976–1995)

After more than half of century of increasingly effective fire control, the Royal Commission on Forest Resources in 1976 acknowledged that “*controlling fire and other* ***natural*** *forces*” was permanently changing ecosystems in BC (Pearse, pg. xv, emphasis added). This new worldview of fire as natural, which recognized “*the historical relationship between fire and the major ecosystem types*” (Province of BC 1981, pg. 107), emerged through the lens of enhanced wildlife protection, advances in ecosystem classification in BC, and a paradigm shift in ecological theory that acknowledged disturbance as integral to ecosystem functioning (Hagerman et al. 2010; Turner 2010; Coogan et al. 2020). Reflecting this worldview shift, a new objective of reintroducing fire was added.

Within the Ministry of Forests, balancing fire control and reintroduction was perceived as straightforward because decision-making authority ultimately rested with the Ministry of Forests district manager who was a “*specialist in a smaller geographic area*” (BCWS #6). For example, prescribed fires were a key strategy that helped reintroduce fire for ecosystem benefits and control future fires by removing hazardous fuels. The Ministry of Forests also worked closely with other actors to reintroduce fire, including the “*forest, ranching and wildlife industries…and the Ministry of Environment*,” who retained authority over wildlife and parks (Province of BC 1989, pg. 15). Research partnerships with the Canadian Forest Service helped advance computer-assisted decision-making for “*whether or not to let a wildfire burn*” based on fire behavior and known values, a strategy known as modified response or managed fire that continues today (Province of BC 1980, pg. 87; Tymstra et al. 2020). The Ministry of Forests also helped the forest industry develop silvicultural strategies to emulate the effects of fire on forest structure. For example, fire was called the “*natural counterpart of clear-cutting*,” justifying clear-cutting as a primary forest management technique (Pearse 1976, pg. 281). Finally, because reintroducing fire contrasted the fire control objective, public education campaigns evolved to “*explain the importance of fire and fire management to forest and range resources*” (Province of BC 1983, pg. 14).

Despite the emphasis on reintroducing and emulating natural fire, the BC Wildfire Service continued to focus on its fire control objective, self-identifying as a “*world leader in fire control and suppression*” (Province of BC 1994). The Ministry of Forests Act (1979) required BC Wildfire Service to set a fire control target for maximum area and volume of timber burnt annually. This target, and the half-century of “successful” fire control, created and perpetuates a dangerous expectation that complete fire control is possible: “*[The public] expect government to be able to do everything they can to save us with suppression, and the reality is, we’re never going to be able to suppress Mother Nature*” (NGO #18).

By the end of the *Emulating Fire* era, the objective of reintroducing fire diminished in priority as concerns grew over wildland-urban interface (WUI) fires, smoke, and climate change. The 1994 wildfire season, which was “*marred by the tragic loss of 18 homes in B.C.'s worst interface fire ever*” (Province of BC 1995), foreshadowed the emergence of catastrophic fire seasons as a key driver of change during the next era and catalyzed an organizational shift that disconnected the BC Wildfire Service from the Ministry of Forests.

### Siloing fire (1995–2017)

In this era, the worldview of fire as natural continued but was balanced against the worldview of fire as unwanted where it negatively affected values: “*Fire is a natural and essential ecological process…however, it can also have undesirable social and economic impacts*.” (Canadian Council of Forest Ministers 2005, pg. 1). From the BC Wildfire Service perspective, the ability to prevent undesirable impacts was constrained by a lack of provincial oversight that limited personnel from traveling outside their district since only “*the district manager could determine if people could respond or not*” (BCWS #13). To remedy this lack of provincial oversight, the BC Wildfire Service became a stand-alone organization in 1995 under the Ministry of Forests and was no longer embedded at the district level. Referred to as the “*big divorce*” (NGO #18, BCWS #12), this separation divided fire and forest actors into silos with contrasting worldviews and objectives of fire: “*the forest sector went and did forestry and the fire people went and did fire*” (BCWS #10). These organizational silos created uncertainty over the responsibility of different actors to address a key driver of change during this era: catastrophic fire seasons.

The “2003 Firestorm” was a catastrophic fire season that forced evacuation of over 45,000 people and demonstrated that relying on fire control was inadequate: “*There was never a fire that didn’t have enough resources. And then all of a sudden, in 2003, we had more fire than we had resource availability*” (BCWS #6). A subsequent independent provincial review into the causes and consequences of the 2003 Firestorm (the Filmon Report) acknowledged that the “success” of fire control since 1912 contributed to the widespread negative impacts. Another contributing factor was the silos created by the “big divorce,” after which “*decisions [were] made by one group without necessarily considering the implications for the other*” (Filmon 2004, pg. 32). Ultimately, the Filmon Report recommended overcoming these silos through a focus on proactive objectives and sharing responsibility with actors that had not yet been part of fire decision-making processes.

A key new actor during this era was First Nations and local communities who were acutely vulnerable to WUI fires. One strategy to support communities was a new funding source, administered by new actors in fire, the Union of BC Municipalities and the First Nations Emergency Services Society (FNESS). FNESS “*had a seat at the table right from the [funding] design*” (NGO #19), and their inclusion mirrored the start of a slow and ongoing shift in BC towards re-instating First Nations’ decision-making power and sovereignty over land (e.g., *Delgamuukw* v British Columbia, 1997 3 SCR 1010). Despite many barriers faced by communities successfully accessing the funding (Ravensbergen et al. 2020; Copes-Gerbitz et al. 2020), the new funding was an important foundation for decision-making authority by communities that had not existed since colonial dominance of fire that started in the *Harnessing Fire* era.

After the 2003 Firestorm, new legislation helped actors understand their roles and responsibilities undertaking multiple fire objectives. First, the Wildfire Act and Regulations (2004) aimed to improve accountability and clarify liability, especially for forest industry actors. This legislation intended to provide “*greater regulatory freedom for the forest industry*” (Province of BC 2003, pg. 42). In doing so, however, it created more “*gray areas*” where the BC Wildfire Service had less authority over strategies such as logging and slash removal that supported fire prevention objectives (BCWS #13). Second, the BC WUI Consequence Management Plan was signed by multiple provincial actors including the BC Wildfire Service, Ministry of Forests, the Provincial Emergency Program, the Office of the Fire Commissioner, Emergency Management BC, the Ministry of Public Safety, and the Solicitor General. This agreement aimed to guide prevention and suppression objectives in the WUI but blurred lines of responsibility among the signatories.

During this era, the BC Wildfire Service underwent a “*strategic shift*” to prioritize proactive objectives and overcome the silos that were created by the “big divorce” (Province of BC 2010, pg. 4). Nevertheless, subsequent catastrophic fire seasons demonstrated that the shift was “*slower and more costly than originally envisioned*” (Canadian Council of Forest Ministers 2016, pg. 5), with “*disappointingly little progress on the goal of enhanced community safety*” (Abbott and Chapman, 2018, pg. 7). For the BC Wildfire Service, these subsequent seasons became an inflection point from which a new vision could emerge: “*we knew our connection with communities, with industry, and internally was starting to be strained and distant…we finally hit the inflection point in’17 and’18 where we didn’t have the relationships and it created a whole bunch of tension at the worst possible time*” (BCWS #12).

### Coexisting with fire (2017 and beyond)

The *Coexisting with fire* era is dynamically emerging in BC in response to ongoing catastrophic fire seasons. Most interviewees attributed 2017 and 2018 to the previous century of fire control objectives: “*we’ve had 100 years of being too good at putting fire out and now we’re living with the legacy*” (BCWS #4). Coupled with climate change that is making the wildfire challenge more complex (BCWS #10), a worldview of fire as inevitable is beginning to take shape. Although many interviewees within BC Wildfire Service “*knew it was a matter of time*” (BCWS #3) until a catastrophic fire season occurred, “*the perspective of [the Ministry of Forests] and the forest industry was significantly shifted to recognizing that those kind of fire seasons back-to-back are…part of our world moving forward*” (BCWS #10). Similarly, interviewees from the Cariboo noted a marked shift in public perception: “*it opened people’s eyes as to the real risk out there; having people really running for their lives in some cases*” (MFLNRORD #7). In 2019, one interviewee speculated on a future in which the inevitability of fire was not recognized, a fear that became a reality during the 2021 fire season, when two lives were lost after nearly 90% of the community of Lytton was destroyed:

“*if things haven’t changed after [2017 and 2018] drastically in how we manage fire, what’s it going to take? One, a community burning down and two, fatalities. Because really, 2017 and 2018 were mind-blowing seasons*” (Expert #1).

These ongoing tragedies have prompted an emerging vision for coexisting with fire that is a focal point for potential transformative change in fire governance: interviewees and recent documents agree that community actors need to have more involvement. The BC Wildfire Service acknowledged that “*following 2017 there was the opportunity to focus around external engagement*” (BCWS #8). Similarly, the independent provincial review into the 2017 fire season highlighted that “*the overwhelming majority of respondents indicated a need to incorporate local knowledge…into wildfire planning*” (“2018 Fire Review” Abbott and Chapman 2018, pg. 59). Community involvement is crucial not only because it helps develop the “social license” needed to enable proactive objectives (BCWS #4, BCWS #8, NGO #15, Copes-Gerbitz et al. 2020) but also because it helps to recognize that “*expertise is everywhere*” (BCWS #6). Supporting this external engagement by the government could involve additional funding for communities to achieve fire prevention objectives; however, interviewees agreed that funding (at the time of interviewing) is inadequate to meaningfully address the inevitability of wildfire (NGO #15) and must be increased from several million dollars to “*several billion dollars over the next 5–10 years*” (Expert #17).

Collaborating with First Nations communities as “*partners and leaders*” is a central component of the proposed vision for coexisting with fire (Abbott and Chapman 2018, pg. 81), especially given BC’s legal commitment to the United Nations Declaration on the Rights of Indigenous Peoples. However, the ideal form of collaboration varied across interviewees, from “*working with them directly through funding programs*” (BCWS #10) to actually sharing power through “*joint-decision making*” (MFLNRORD #7). Several interviewees from the BC Wildfire Service agreed that a primary pathway for increased decision-making by First Nations is reintroducing cultural fire from the *Harnessing Fire* era in a form of reimagined stewardship: “*First Nations want to bring burning back…[They] are holding a lot of political power in the province right now…and I think that’s probably the biggest collaboration to help move things forward*” (Expert #3).

While all interviewees agreed that communities are central actors for enabling proactive objectives, opinions varied as to the spatial scale at which proactive objectives should occur. Some advocated that FireSmart initiatives to create defensible space around the home and in the WUI are “*the best tool we can use to help public safety*” (BCWS #3). Others stressed that community protection requires fire risk at the landscape level where many communities’ livelihoods are drawn from. Across all scales, community input is key to decision-making of potential trade-offs: “*[The public] says ‘we don’t want another season like 2017,’18,’ so what does it look like to avoid that?*” (BCWS #13). Both the BC Wildfire Service and Ministry of Forests have committed to prioritizing proactive objectives; as of 2020, the BC Wildfire Service reports a new target of “*improve community resilience through proactive and collaborative hazard management*” (Province of BC 2020, pg. 10). This target is reflected in the Cariboo Region Ministry of Forests vision, where fire is now a central theme of “*vibrant, connected and resilient communities*” (MFLNRORD #9).

Especially for interviewees outside of the BC Wildfire Service, the vision for coexisting with fire requires reconnecting fire to forest governance by implementing a landscape-level planning process. One interviewee described the ideal planning process as “*adaptive management, [where we] engage the public, recognize our goals and objectives and set out strategies and implement, learn, monitor, adjust, and go forward*” (NGO #2). Collaborative planning processes that are currently being piloted (including one in the Cariboo) aim to contrast historical efforts that were centralized and top-down: *“this isn’t the same old government process that’s been pushed down our throats for 100 and something years. This is something new…we’re actually listening*” (MFLNRORD #7). Perhaps contrarily, interviewees agreed that planning should be initiated by the Ministry of Forests because they are “*experts in land management*,” with input from the BC Wildfire Service who are “*experts in fire*” (NGO #11). A primary component of the planning process must be to explicitly consider how fire interacts with broader decision-making on forests: “*mitigating wildfire risk must now be a key mandate in all land use planning and activities*” (NGO #11).

Such a mandate must be supported with legislation because “*what really drives change is a requirement in legislation…that is crafted [with] the involvement of key stakeholders*” (NGO #11). The responsibility for introducing legislative change lies with both the BC Wildfire Service and the Ministry of Forests, but the pathway for “*involvement of key stakeholders*” was not articulated. Both the interviewees and the 2018 Fire Review (Abbott and Chapman 2018) recommended adding fire as a stated value in the Forest and Range Practices Act (FRPA), which would require forest professionals to meet fire risk reduction goals “*in a community where there are multiple values; it’s not about fire over everything*” (BCWS #10). Furthermore, it could support landscape-level fire prevention objectives by “*directing industry in a compliance way to [undertake strategies] on the landscape that benefit wildfire risk reduction*” (BCWS #13). Legislative changes could also help overcome challenges with the “professional reliance” model of forestry in BC that some interviewees felt was disconnected from fire prevention objectives (BCWS #3, #12). Since 2017, there has been a strong push within BC Wildfire Service to “*continue driving policy change to keep up [as] conditions in society and values change*” (BCWS #4). Impending changes to FRPA and the Wildfire Act and Regulations will show whether this has been the case.

In addition to external engagement and legislative changes, interviewees’ vision for coexisting with fire includes “*bridging the disconnect between land management and fire that [resulted] from a culture of [fire] suppression*” (Expert #1). Progress towards overcoming silos created during the big divorce is ongoing, following a recommendation from the 2018 Fire Review that the BC Wildfire Service be “*operationally reintegrated into [Ministry of Forests] regional operations*” (pg. 94). Currently, the BC Wildfire Service and Ministry of Forests (at the district level) are sharing personnel through cross-training, collaborating on fire prevention projects, and facilitating collaborative landscape planning processes. One interviewee noted the importance of training in both fire and forestry to have a “*better idea of managing the landscape and how making decisions on fires might impact forest management*” (Expert #5).

Collectively, through transformative change that incorporates First Nations and local community actors into decision-making processes, updates legislation, and reintegrates the Ministry of Forests and BC Wildfire Service to lead landscape-level planning, interviewees were hopeful that the diverse actors, worldviews, and objectives of fire can coexist: “*this is a shared responsibility, from the provincial government all the way down to the individual homeowner…That's the only way we're going to solve it because it is so complex*” (BCWS #10).

## Discussion

Our historical analysis reveals three key insights for understanding the future of fire governance in BC. First, interviewees and recent strategic documents largely share a vision for a model of fire governance (coexisting with fire) that includes greater involvement of diverse, non-government actors. Second, achieving this vision must address two primary constraints on change, organizational rigidity and siloed expertise, that result from entrenched colonial power. Third, overcoming these constraints requires intentional intervention by both the BC Wildfire Service and Ministry of Forests to ensure that transformation is also defined and led by First Nations.

### The emergence of a multi-actor vision for future fire governance

The vision for coexisting with fire put forward by interviewees identifies the importance of increasing the decision-making power of First Nations and local communities, and a need for a more direct support on fire prevention from the forest industry. This vision aligns closely with provincial (Abbott and Chapman 2018; Daniels et al. 2018) and federal (Sankey 2018; Tymstra et al. 2020) calls for transformation in response to the twenty-first century catastrophic fire seasons in Canada. In contrast to a century of government-dictated worldviews and objectives, this vision would seek to incorporate multiple worldviews of fire (beneficial, natural, unwanted, and inevitable). Cohesive agreement on this vision suggests an increasingly unified understanding of the need for diverse views to address the complex fire problem (Tedim et al. 2019; Tymstra et al. 2020), an important condition for enabling transformation (Chaffin et al. 2016; Schultz et al. 2019).

A primary pathway to facilitate transformation is sharing decision-making with communities to enable proactive objectives (Filmon 2004; Abbott and Chapman 2018). Examples of strategies to encourage decision-making include implementing the seven disciplines of FireSmart (education, vegetation management, emergency planning, cross-training, interagency cooperation, legislation and planning, and development; BC FireSmart Committee 2022) and input into forest management planning more broadly. The need for community expertise in planning was echoed in the adjacent province of Alberta after the 2016 catastrophic fire season (Sherry et al. 2019). Despite calls for shared decision-making, however, experiences in BC since the 2003 Firestorm (BC Forest Practices Board 2015; Ravensbergen et al. 2020; Dickson-Hoyle and John, 2021; Copes-Gerbitz et al. 2022) mirror that of Australia (Lukasiewicz et al. 2017; Reid et al. 2018) where the rhetoric of “shared responsibility” does not necessarily amount to meaningful devolution of decision-making power with supports to address persistent capacity barriers. Therefore, while sharing decision-making is a critical step towards transformation, the paucity of social science research to understand the unique expertise of First Nations and local community actors, and pathways for empowerment, remains a key knowledge gap in BC (Abbott and Chapman 2018; Lewis et al. 2018; Sankey 2018; Sherry et al. 2019; Steelman et al. 2021).

The emerging vision for coexisting with fire also requires a greater role for the forest industry because they can proactively address fire risk at the landscape scale (Lieffers et al. 2020). Given a long history of industry power in forest and fire management, however, interviewees and documents agreed that forest industry involvement needs to be mandated. Interviewees advocated for somewhat more prescriptive legislation and guidelines, such as the Wildfire Act and Regulations, the Forest and Range Practices Act, and the Hazard Abatement Guidelines. Mandatory, rather than voluntary, accountability is key for compliance of market-based actors in transformative change (Lemos and Agrawal 2006). Revised legislation with clearer proactive objectives could also enhance the current model of professional reliance of industry actors. This model can be problematic because short-term profit maximization is often the primary objective of the forest industry in BC (Mitchell et al. 2017; Haddock 2018). Ultimately, any legislation that addresses and influences fire must help industry (and other) actors navigate short- vs. long-term tradeoffs of different objectives (e.g., fire suppression vs. reintroducing fire) in a way that prioritizes proactive objectives (Steelman 2016; Schultz et al. 2019; Tymstra et al. 2020).

### Organizational rigidity and siloed expertise constrain change

Transformation in BC fire governance is currently constrained by organizational structures that arose from entrenched government power. Centralizing organizational power constrains transformation because the actors involved tend to favor the status quo (Farrelly and Brown 2011; Chaffin et al. 2016). In Canada (Sherry et al. 2019; Tymstra et al. 2020) and the USA (Moseley and Charnley 2014; Schultz et al. 2019), rigid, control-based fire organizations are the norm. The historical lens demonstrates that organizational rigidity in the Ministry of Forests and BC Wildfire Service resulted from power accumulating through time, as colonial worldviews of fire became institutionalized in legislation, objectives, strategies, and decision-making processes (Mathevet et al. 2015). This power is a result of both internal and external factors (Steelman and McCaffrey 2011): the internal “*culture of suppression*,” reinforced through fire suppression training and government targets (Expert #1, *Coexisting with Fire* era) and the external public expectation of suppression effectiveness (NGO #18, *Emulating Fire* era). Organizational rigidity is mirrored in the broader forest management context in BC: although the Ministry of Forests retains responsibility for forest management, the forest industry continues to wield decision-making power (Kamieniecki 2000), evidenced by the entrenched worldview of fire as destructive to timber values that started in the *Controlling Fire* era.

Silos of expertise in colonial environmental governance are also pervasive because of historical processes of centralization (Cumming et al. 2006) and the exclusion of other forms of expertise, such as Indigenous knowledge (Pelai et al. 2021). Siloed colonial expertise is a primary constraint on transformational change in fire governance (Smith et al. 2016; Steelman 2016; Schultz et al. 2019; Tedim et al. 2019) and the division between fire and forestry in the BC government is a prime example of this. Mirroring siloed expertise in Canada more broadly (Hirsch et al. 2001), in BC, it resulted from the active suppression of Indigenous fire stewardship in favor of Western forestry expertise and the “big divorce” of 1995 when fire control and forest management expertise were separated in the BC Wildfire Service and the Ministry of Forests, respectively. Recognizing the gaps in connected and Indigenous expertise, the BC Wildfire Service is actively supporting the development of fire competencies for professional foresters and seeking ways to better incorporate Indigenous expertise in decision-making processes. This intention is critical given the important role of individual actors in institutionalizing change (Moseley and Charnley 2014), but it was the 2017 catastrophic fire season that catalyzed efforts by the government to address entrenched power.

### Overcoming constraints to enable transformation

The 2017 fire season demonstrated that governance-as-usual is inadequate in BC and opened an opportunity for transformation towards coexisting with fire, which has become a mantra for fire researchers globally (Moritz et al. 2014; Roos et al. 2016; Schoennagel et al. 2017; McWethy et al. 2019; Tedim et al. 2019). Critically, however, transformation requires intentional intervention by powerful organizations to alter rigid and path-dependent structures that are constraining change (Folke et al. 2010; Westley et al. 2011; Chaffin et al. 2016; Hahn and Nykvist 2017; McWethy et al. 2019). Interviewees spoke of intentional changes by the BC Wildfire Service and the Ministry of Forests to overcome their internal silos and address power imbalances by focusing on external engagement. Nevertheless, the slow and incomplete changes after the 2003 Firestorm meant some interviewees were skeptical about how transformational proposed changes would be. In fact, ongoing challenges experienced by First Nations enacting their decision-making authority in subsequent years (Verhaeghe et al. 2019; Dickson-Hoyle and John 2021; Hoffman et al. 2022) suggest that transformation has not yet meaningfully occurred for these communities.

Ensuring that transformation *is defined and led by* First Nations (Sankey 2018; Lam et al. 2020) is key for adhering to the new legislation in BC applying the United Nations Declaration on the Rights of Indigenous Peoples. Equal partnerships with First Nations would address existing objectives and strategies (e.g., modified response or evacuations) that are implemented based on non-Indigenous priorities and negatively affect Indigenous communities (Abbott and Chapman 2018; McGee et al. 2019; Zahara 2020). Furthermore, reintroducing cultural fire as a primary objective provides an opportunity for First Nations peoples to “reassert” their knowledge (Martello and Jasanoff 2004; Armitage et al. 2012) by holding decision-making authority. This authority is key for transformation because fire-related objectives are intimately playing out on Indigenous peoples’ unceded traditional territory (Wilson 2019; Caverley et al. 2020) and they have their own expertise of fire (Nikolakis and Roberts 2020). First Nations-led or co-governance models could share decision-making among actors (Wyatt 2008), such as the BC Community Forestry model that successfully enhances community resilience to fire (Ambus and Hoberg 2011; Copes-Gerbitz et al. 2020; Devisscher et al. 2021). Ultimately, supporting the vision of coexisting with fire requires devolution of entrenched government power, and appropriate community supports, to ensure transformation is equitable (Offen 2004; Nayak et al. 2016; Bennett and Satterfield 2018).

## Conclusion

Transformative change to coexist with fire requires organizations, policy-makers, and communities to explicitly consider which actors, objectives, and worldviews influence land governance in BC (Stojanovic et al. 2016; Caverley et al. 2020). Changing fire governance by the BC Wildfire Service will not result in transformation unless land governance by the Ministry of Forests and the province of BC changes simultaneously. In BC, one mechanism for such change is a Forestry Commission that intimately considers the interactions between land governance and fire. The last substantial Commission occurred in 1976 and coincided with a new era of fire governance — but was still based largely on colonial priorities. A new Forestry Commission that is led by a First Nations commissioner with input from government and other relevant stakeholders could leverage revelations from the catastrophic fire seasons in 2017, 2018, and 2021 to articulate a vision beyond that which is presented here and outline the concrete pathways for coexisting with fire in BC.

## Footnotes

1 The Constitution of Canada recognizes three groups of Indigenous peoples, including First Nations, Métis, and Inuit. First Nations were the primary land holders prior to colonization in BC, so we use First Nations unless specific sources or statements refer to Indigenous peoples more broadly.

2 The BC Wildfire Service is the organization name as of publication. We use BC Wildfire Service throughout; however, it has undergone multiple name changes through time, starting as the Forest Protection Division (1912), then the Protection Branch (1978), then the Wildfire Management Branch (2010).

3 We use the definition of “wildfire management” from the Canadian Wildland Fire Management glossary (2017) which encompasses all activities relating to wildfire prevention, preparedness, response, and recovery objectives.

4 The Ministry of Forests, Lands, Natural Resource Operations, and Rural Development (Ministry of Forests) is the organization name as of publication. We use Ministry of Forests throughout; however, it has undergone multiple name changes through time, starting as the Forest Branch (1912), then the Forest Service (1945), and was elevated to a ministry in 1978.
